# Items

**Published:** 1874-07

**Authors:** 


### ITEMS.

Dr. E. S. Gaillard, has entered upon the publication of a new medical Journal entitled *The American Medical Weekly.* He still continues the publication of the *Richmond and Louisville Medical Journal*, in the editorial management of which he has gained a wide reputation. We wish him an equal measure of success in his new undertaking.——*The Northwestern Medical and Surgical Journal* has been discontinued.——Dr. Wm. A. Hammond has again revived the *Psychological Journal* under the name of the *Psychological and Medico-Legal Journal.* He is assisted in the editorial management by Dr. T. M. B. Cross. The Journal is published by F. W. Christern, New York.——We have received the Annual Announcement of the Buffalo Medical College, for the session of 1874–75. The present announcement contains a list of the Alumni of the College with their residences, as far as could be ascertained, together with the address of Joseph Warren, Esq., at the last commencement. We notice an important change in the course for the coming session. During the preliminary term lectures will be delivered in the College ampitheatre by Professors White, Hadley, Potter and Stoddard. There will also be clinical lectures as usual by Professors Rochester and Miner, at one of the Hospitals. This is an important addition to the course of lectures, and will give the students an insight into some preliminary subjects which cannot be considered in the regular course.——Dr. James G. Hyndman has assumed the duties of Assistant Editor of the *Clinic.*——Mr. Wm. H. Peabody has issued a price list of Surgical Instruments and appliances for sale by him. It contains also full directions for taking measurements for Hip-Joints, splints, spinal supporters, and other apparatus for deformities. It will be sent to any physician on application.

